# In-Plane Anisotropy of Electrical Transport in Y_0.85_Tb_0.15_Ba_2_Cu_3_O_7−x_ Films

**DOI:** 10.3390/ma17030558

**Published:** 2024-01-24

**Authors:** Matvey Lyatti, Ines Kraiem, Torsten Röper, Irina Gundareva, Gregor Mussler, Abdur Rehman Jalil, Detlev Grützmacher, Thomas Schäpers

**Affiliations:** 1 Peter Grünberg Institut (PGI-9), Forschungszentrum Jülich, 52425 Jülich, Germanyth.schaepers@fz-juelich.de (T.S.); 2 JARA-Fundamentals of Future Information Technology, Jülich-Aachen Research Alliance, Forschungszentrum Jülich and RWTH Aachen University, 52425 Jülich, Germany; 3 Peter Grünberg Institut (PGI-10), Forschungszentrum Jülich, 52425 Jülich, Germany; a.jalil@fz-juelich.de

**Keywords:** high-temperature superconductivity, thin-film, in-plane anisotropy, superconducting transition

## Abstract

We fabricated high-quality c-axis-oriented epitaxial YBa_2_Cu_3_O_7−x_ films with 15% of the yttrium atoms replaced by terbium (YTBCO) and studied their electrical properties. The Tb substitution reduced the charge carrier density, resulting in increased resistivity and decreased critical current density compared to pure YBa_2_Cu_3_O_7−x_ films. The electrical properties of the YTBCO films showed an in-plane anisotropy in both the superconducting and normal states that, together with the XRD data, provided evidence for, at least, a partially twin-free film. Unexpectedly, the resistive transition of the bridges also demonstrated the in-plane anisotropy that could be explained within the framework of Tinkham’s model of resistive transition and the Berezinskii–Kosterlitz–Thouless (BKT) model, depending on the sample parameters. Measurements of the differential resistance in the temperature range of the resistive transition confirmed the occurrence of the BKT transition in the YTBCO bridges. Therefore, we consider the YTBCO films to be a promising platform for both the fabrication of devices with high kinetic inductance and fundamental research on the BKT transition in cuprate superconductors.

## 1. Introduction

Superconducting films with a high kinetic inductance are considered a promising platform for many quantum applications, including quantum computing and quantum communication. Kinetic inductance (*L_K_*) arises from the kinetic energy stored in the motion of charge carriers rather than the energy stored in a magnetic field. The impedance of nanoscale superconducting devices and Josephson junctions, where the kinetic inductance of a supercurrent dominates at operating frequencies, can achieve very high values. Currently, high-kinetic-inductance devices are based on disordered low-temperature superconductors such as NbN [1,2,3], NbTiN [4], NbSi [5], natural Josephson weak-link arrays formed in granular aluminum [6], artificial objects such as Josephson junction arrays [7,8], or semiconductor structures with induced superconductivity [9]. An alternative approach to high-kinetic-inductance materials for quantum applications could be based on high-temperature cuprate superconductors, where a fully gapped state is created by finite-size effects, reductions in the doping level, or phase fluctuations [10,11,12,13,14,15,16]. Cuprate superconductors have low charge carrier densities and, hence, they have high kinetic inductance, which can be calculated as follows:

*L_k□_* = *m_e_*/2*de*^2^*n_s_* = *ħρ_n_*/Δπ*d* = *μ*_0_*λ*^2^/*d*,

where *m_e_* is the free electron mass, *e* is the electron charge, *n_s_* is the superfluid density, *μ*_0_ is the permeability of the free space, *ħ* is the Planck constant*, ρ_n_* is the normal-state resistivity, *λ* is the magnetic field penetration depth, and *d* is the film thickness [17,18]. The in-plane magnetic penetration depth *λ* of YBa_2_Cu_3_O_7−x_ (YBCO) is comparable to values that have been reported for NbN films. Further increases in the kinetic inductance of YBCO films without creating oxygen vacancies are possible by the substitution of yttrium (Y) with another rare-earth metal [19,20]. Among the different rare-earth metals, terbium (Tb) is known for its ability to increase the in-plane resistivity of YBCO by more than one order of magnitude without reductions in the critical temperature, for a Tb content up to 50% [21,22,23,24]. Since kinetic inductance is proportional to resistivity, the high resistivity might be a sign of the high kinetic inductance of Tb-substituted YBCO films. However, the exact mechanism for the increase in the resistivity of YBCO with increasing Tb content is not clear. On the one hand, the resistivity increase has been attributed to the Tb being in a mixed-valence state that can reduce hole doping [21]. On the other hand, the mixed-valence state of Tb has not been confirmed by neutron diffraction and X-ray data [25].

We fabricated high-quality Y_0.85_Tb_0.15_Ba_2_Cu_3_O_7−x_ (YTBCO) films and studied their electrical properties. We found that the YTBCO films possess in-plane anisotropy in both normal and superconducting states that, together with X-ray diffraction (XRD) data, provides evidence for a twin-free film. The in-plane anisotropy of the resistive transition could be explained within the framework of Tinkham’s model of resistive transition [26] and the Berezinskii–Kosterlitz–Thouless (BKT) theory, depending on the sample parameters [27]. By measuring the Hall effect in the YTBCO as well as in the pure YBCO films, we showed that the increased resistivity in the YTBCO films was due to the decrease in the charge carrier density.

## 2. Experimental Details

We deposited the epitaxial YBCO films with 15% of the Y atoms replaced with Tb on (100) SrTiO_3_ substrates by DC sputtering of a Y_0.85_Tb_0.15_Ba_2_Cu_3_O_7−x_ target at a high oxygen pressure of *p*(O_2_) = 3.4 mbar. The substrate edges were aligned along the (010) and (001) planes. The temperature of the substrate heater was 935 °C. The surface of the (100) SrTiO_3_ substrates had a TiO_2_ surface termination resulting from etching in a buffered oxide etch. The film thickness *d* was controlled by the sputtering time. The deposition rate of 1.38 nm/min was calibrated with X-ray reflectometry (XRR) and profilometer measurements of the films’ thicknesses. After deposition of each film, the heater temperature was ramped down to 500 °C at a rate of 30 °C/min, and then the deposited films were annealed for 15–30 min in pure oxygen at a pressure of 800 mbar. Following the annealing, the films were cooled down to room temperature at a rate of 30 °C/min. The films thinner than 21 nm were additionally protected by a 7 nm thick, non-superconducting, amorphous YTBCO layer deposited in situ at a 50–60 °C heater temperature, which was required to protect the films during structuring. The oxygen doping in the films with the amorphous YTBCO layer on top may have been at a lower level than that in the films that did not possess a protective layer due to the plasma heating during the deposition of the amorphous YTBCO layer. No additional oxygen annealing was performed to avoid increasing the surface roughness of the amorphous layer. In addition to the YTBCO films, we deposited two reference YBCO films for Hall effect measurements using the same technique.

**Figure 1:**
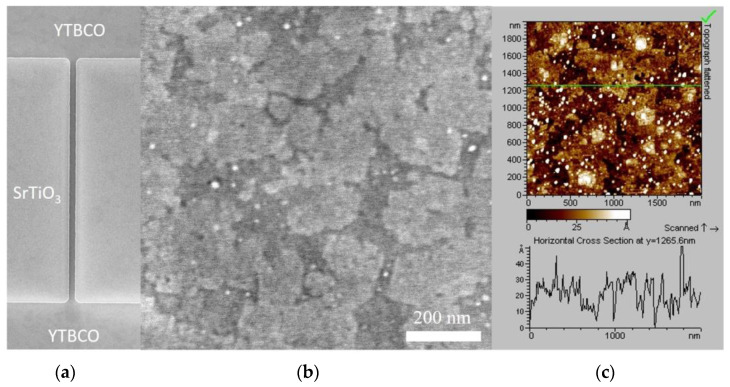
SEM image of the 100 μm long YTBCO bridge with the measured width of 3 μm (**a**). Images of the YTBCO film surface obtained with scanning electron (**b**) and atomic force microscopy. A green line shows the cross-sectional position (**c**).

The YTBCO films were patterned into long bridges oriented along the [100] and [010] substrate crystallographic axes by contact UV lithography and chemical wet-etching in a Br–ethanol solution. The bridges patterned in the PMMA resist along one crystallographic direction had widths (*W*) of 3, 5, 10, and 20 μm and, along another crystallographic direction, widths of 5, 10, and 20 μm, with a length (*L*)-to-width ratio of *L*/*W* = 20. The YTBCO bridges were 1–2 μm narrower than the corresponding PMMA resist patterns because of the undercut during wet chemical etching. The actual bridge widths were determined by scanning electron microscopy (SEM) measurements. A representative SEM micrograph of a 3 μm wide and 100 μm long bridge is shown in Figure 1a.

Current–voltage (*IV*) characteristics of the current-biased bridges were measured in a zero magnetic field by a four-probe technique using self-made low-noise battery-driven electronics. The temperature dependence of the bridge resistance was obtained with a lock-in amplifier at a 10 kHz modulation frequency. The Hall measurements were performed in DC magnetic fields up to 1 T using current and magnetic field reversal.

## 3. Experimental Results and Discussion

### 3.1. Structural Analysis

We fabricated several YTBCO films with thickness values in the 11.8–123 nm range on SrTiO_3_ substrates. Representative images of a film surface obtained with an SEM and an atomic force microscope (AFM) are shown in Figure 1b,c. The films demonstrated layer-by-layer growth similar to that achieved for YBCO films in our previous work [28]. The contrast between the darker and lighter areas in the SEM image corresponded to the thickness difference of one unit cell (u.c.). The AFM micrograph showed that the surface of the YTBCO films had a root-mean-square roughness of 1.04 nm with round-shaped nano-precipitates with a diameter of 10–20 nm and a height of a few nm.

**Figure 2:**
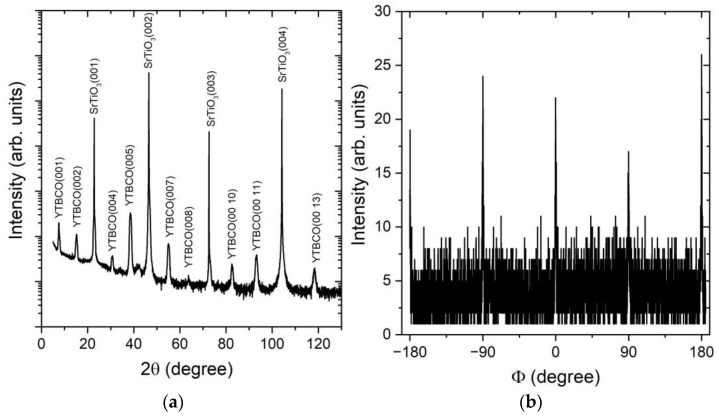
XRD 2Θ-Θ (**a**) and Φ (**b**) scans of a 35 nm thick YTBCO film (sample N5).

**Figure 3:**
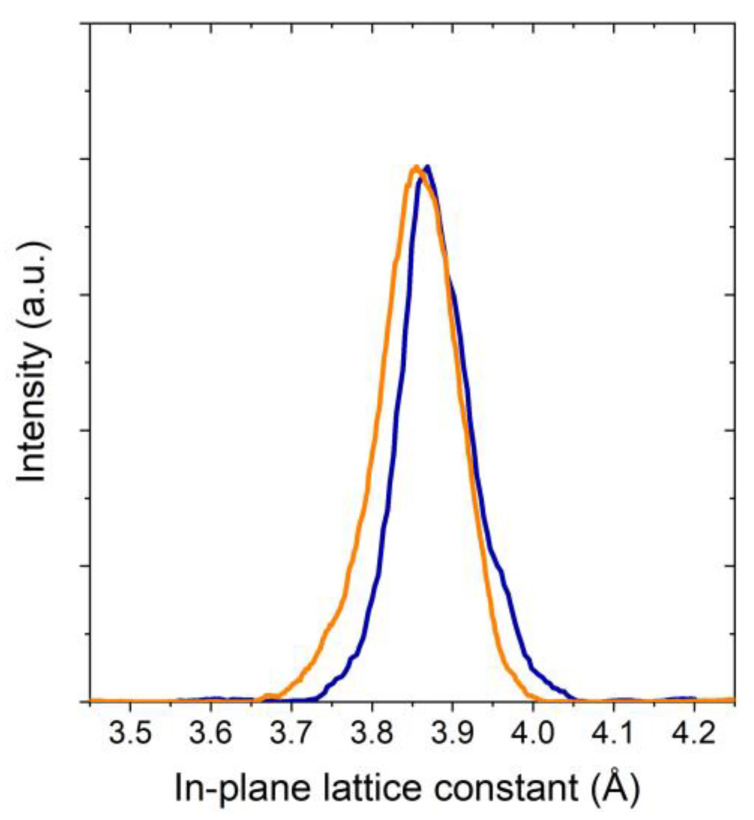
Cross sections of reciprocal space maps (RSM) around the (108) (orange curve) and (018) (blue curve) reflections.

Since it has been shown that the formation of secondary phases is possible when the Tb content exceeds 30% [22,29], we investigated the in-plane and out-of-plane crystallographic properties of our YTBCO films by XRD analysis with a high-resolution Bruker D8 Discover diffractometer. A 2Θ-Θ scan of a 35 nm thick YTBCO film is shown in Figure 2a. Only (00I) YTBCO reflections were observed in the 2Θ-Θ scan, indicating single-crystalline growth with the *c* axis in the growth direction. No secondary phases were seen. From the angular position of the (00I) peaks, we calculated the length of the YTBCO *c*-axis lattice parameter to be 11.65 Å. The *a*- and *b*-axis lattice parameters were 3.86 Å and 3.87 Å*—*measured by means of reciprocal space maps (RSM) around the (108) and (018) reflections using a high-resolution Rigaku Smartlab diffractometer. The RSM cross sections presented in Figure 3 show two distinct peaks related to the *a*- and *b*-axis lattice parameters of the YTBCO film. The small difference between the *a*- and *b*-axis lattice parameters, resulting in overlapping RSM peaks, did not allow us to draw a quantitative conclusion about the degree of film twinning based on the XRD data alone. We examined the films using polarized light optical microscopy and found no evidence of twinning. We attributed the reduced difference between the *a*- and *b*-axis lattice parameters compared to pure YBCO samples to the effect of Tb substitution [30]. The in-plane crystallographic structure was further analyzed by a Φ scan. The Φ scan around (104) and (014) reflections is shown in Figure 2b. The peaks spaced at a periodicity of 90° for both reflections demonstrated the good in-plane order of the film.

### 3.2. Electrical Transport in Normal State

The normal-state resistance of the YTBCO bridges had a metallic temperature dependence with a sharp superconducting transition, as observed for the pure YBCO films [28]. Taking into account the nonconducting layers at the top and bottom interfaces of a YTBCO film [28], we calculated the resistivity as

**Figure 4:**
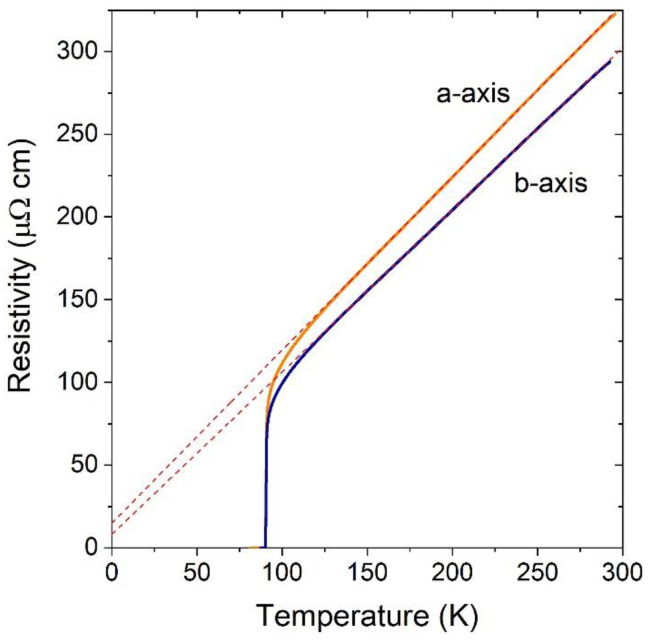
Resistivity temperature dependence of the 8 μm wide and 123 nm thick bridges oriented in *a* and *b* crystallographic directions (sample N7). Dashed lines represent linear fits.

*ρ*_*n*_ = *R*[*W*·(*d* − 2 u.c.)]/*L*,

where *R* is the bridge resistance. The resistivity of the YTBCO bridges oriented along different substrate edges demonstrated a clear in-plane anisotropy of the resistivity, as shown in Figure 4. Here, we assume that the bridges with higher resistivity are aligned in the *a*-axis direction and the bridges with lower resistivity in the *b*-axis direction, as observed for the pure YBCO [31]. The results of the resistivity calculations are presented in Table 1.

We found that the thicker films had a *ρ_n_* (300 K)/*ρ_n_* (100 K) ratio close to 3, which is typical for optimally doped YBCO films, while this ratio for the thinner films with a protective layer was *ρ_n_* (300 K)/*ρ_n_* (100 K) = 2.4–2.7, which is a signature of underdoped films. The details of the *ρ_n_* (300 K)/*ρ_n_* (100 K) ratio calculations are discussed in the Supplementary Materials. The lower oxygen concentration in the thinner films is likely due to the plasma heating during the deposition of the protective layer.

To calculate an in-plane resistivity anisotropy ratio, we used the resistivity values at T = 100 K. We found the in-plane anisotropy of the resistivity to be in the range of 1.05–1.13 for the optimally doped films and 1.08–1.45 for the underdoped films, as shown in Table 1. The measured in-plane anisotropy was smaller than that of the twin-free YBCO samples where it was in the range of 1.6–2.2 [31,32]. The reduced in-plane anisotropy may be related to the reduced difference between the *a*- and *b*-axis lattice parameters due to the Tb substitution [30].

The resistivity of the optimally doped YTBCO films was twice as high as the resistivity of the optimally doped YBCO films deposited with the same technique [28]. To find out the reason for the increased resistivity of the YTBCO films, we measured the Hall coefficients and the Hall mobility for the YTBCO films at room temperature. Since the charge carrier concentration can depend on the deposition technique and oxygen annealing conditions, we also fabricated a pure YBCO film for reference, using the same sputtering and annealing parameters. Measurements were performed with eight-terminal Hall bars and Hall crosses oriented in the *a* and *b* crystallographic directions. The 30 nm thick reference YBCO film showed a room-temperature resistivity of 208 ± 3 μΩ·cm and a Hall coefficient of (7.2 ± 0.1)·10^−4^ cm^3^/C. The YBCO film did not demonstrate in-plane anisotropy due to film twinning. The Hall coefficient of the YBCO film was close to the values reported for optimally doped YBCO single crystals and films [33,34]. In contrast to those of the twinned YBCO film, both the Hall coefficients and the resistivity of the YTBCO films had in-plane anisotropy typical of twin-free YBCO samples [35]. Two representative YTBCO films with average room-temperature resistivities of 307 ± 13 μΩ·cm and 419 ± 19 μΩ·cm showed average Hall coefficients of (9.45 ± 0.05)·10^−3^ cm^3^/C and (1.31 ± 0.06)·10^−3^ cm^3^/C, respectively. Therefore, we concluded that Tb substitution reduces the charge carrier concentration in the YTBCO, as expected in the case of the mixed-valence state of the Tb [21]. The smaller charge carrier concentration favors a greater kinetic inductance which is the goal of this work.

### 3.3. Electrical Transport in Superconducting State

**Figure 5:**
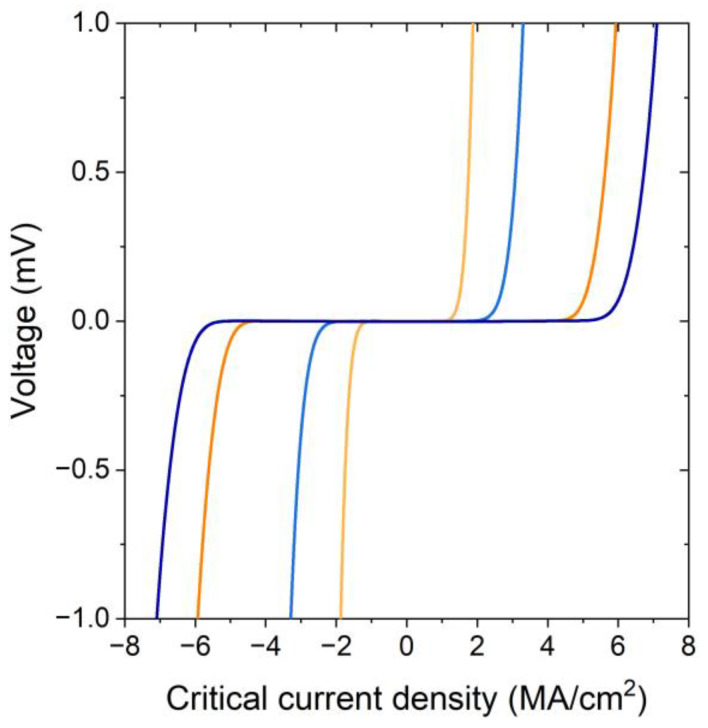
*IV* curves of the 8–9 µm wide bridges placed on samples N1 and N6 and oriented in perpendicular directions. Light blue and light orange curves correspond to sample N1. Dark blue and orange curves correspond to sample N6.

The transport properties of the YTBCO bridges in the superconducting state were studied by measuring the *IV* curves of the bridges at *T* = 77.4 K. Representative *IV* curves of the YTBCO bridges oriented in perpendicular directions are shown in Figure 5 for underdoped (N1) and optimally doped (N6) samples. The *IV* curves demonstrate a typical flux-flow behavior in the resistive state. We determined the critical current values *I_c_* with the 10 µV criterion and calculated the critical current density as *J_c_* = *I_c_*/[*W*·(*d* − 3.u.c.)], taking into account the non-superconducting layer at the YTBCO–substrate interface [28]. The thickness of the “dead” layer in the superconducting state is one unit cell larger than that in the normal state due to the reduced critical temperature of the third unit cell layer, such that it is either non-superconducting at 77.4 K or its superconducting properties have significantly deteriorated compared to the subsequent layers [28]. Thus, a layer with a thickness of three unit cells does negligibly contribute to the superconducting transport of the thin film at *T* = 77.4 K. The calculated critical current densities showed a clear in-plane anisotropy with the ratio *J_cb_*/*J_ca_* in the range of 1.07–1.23 for the optimally doped films and 1.72–1.98 for the underdoped films, where *J_ca_* and *J_cb_* are the critical current densities in the *a*- and *b*-axis crystallographic directions, respectively. The corresponding values are given in Table 1. To confirm the reliability of the critical current density measurements, we fabricated an additional sample where seven bridges with the same width were oriented in the same direction. The 2% standard deviation of the critical current values for these bridges was well below the measured in-plane anisotropy of the critical current density. Therefore, we concluded that the difference between *J_c_* values in the *a*- and *b*-axis directions was due to the anisotropy of the film properties. The in-plane anisotropy of the critical current density of the YTBCO films is close to that reported for partially [36] and completely twin-free YBCO samples [37,38]. We believe that the in-plane anisotropy of both the normal-state and superconducting properties of the YTBCO films together with XRD and optical microscopy data provide evidence that our YTBCO films are at least partially untwinned.

Both the critical current density and the conductivity of the optimally doped YTBCO films were approximately two times lower than those of the YBCO films fabricated with the same technique. Therefore, we expect that the kinetic inductance of the YBa_2_Cu_3_O_7−x_ films, where 15% of the Y atoms are replaced with Tb, is twice as high as in pure YBCO films. A further increase in the kinetic inductance is possible at higher Tb concentrations.

### 3.4. In-Plane Anisotropy of Superconducting Transition

The average midpoint critical temperature *T_c,mid_* of the YTBCO films, corresponding to the middle of the transition, and the superconducting transition width Δ*T_c_* were in the ranges of 87.7–90.1 K and 0.4–2 K, respectively, as shown in Table 1. These values are very close to those for pure YBCO films of the same thickness [28]. The width of the superconducting transition was determined according to a 90–10% resistivity drop criterion. The narrow superconducting transition, which is only 0.15 K broader than the best values reported for YBCO single-crystal samples, confirmed the high quality of the YTBCO films [39].

**Figure 6:**
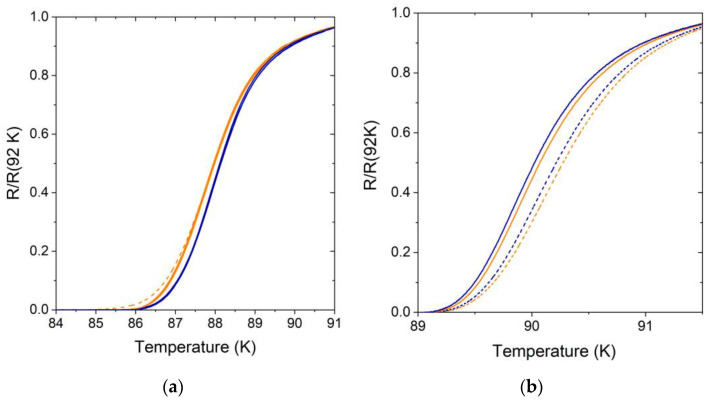
(**a**) The superconducting transition of seven 11.8 nm thick YTBCO bridges (sample N1) with widths ranging from 2 to 19 μm oriented along the (**a**) (orange lines) and (**b**) (blue lines) crystallographic axes. The *R(T)* curve of the narrowest bridge is shown by the dashed line. (**b**) The superconducting transition of four 21 nm thick YTBCO bridges (sample N3) with widths of 19 μm (solid line) and 9 μm (dashed line). The bridges oriented along the *a* and *b* crystallographic axes are shown in orange and blue, respectively.

By measuring the *R*(*T*) dependences, we noticed that the bridges with the same width patterned on the same substrate but oriented along different crystallographic axes had slightly different midpoint critical temperatures which might be a signature of the films’ inhomogeneity or related to the in-plane anisotropy. To study the origin of the critical temperature scattering, we carried out a more thorough study of the superconducting transition, ramping the temperature up and down in the 75–95 K temperature range. We gradually decreased the temperature ramp rate until the temperature hysteresis of the *R*(*T*) dependence became less than the difference between the critical temperatures of the bridges oriented along the different crystallographic axes. In addition, we reduced the AC bias current until it had no measurable influence on the critical temperature value. The final *R*(*T*) dependence curve was calculated as the average of the *ρ*_*n*_(T) curves measured during the ramping temperature up and down. Representative R(T) dependences acquired for seven 11.8 nm thick bridges with widths ranging from 2 to 19 μm on the same substrate are presented in Figure 6a. The seven *R*(*T*) dependencies were divided into two groups. Within each group, *R*(*T*) dependencies matched perfectly. The *R*(*T*) dependencies with higher *T*_*c*_, shown in blue, corresponded to the bridges with higher critical current density, while the *R*(*T*) dependencies with lower *T*_*c*_, shown in orange, corresponded to the bridges with lower critical current density. Since the bridges with higher critical current density had lower normal-state resistivity, we concluded that these bridges were oriented in the b-axis direction, while the bridges with lower critical current density were oriented in the a-axis direction. The higher resistance of the narrowest 2 μm wide bridge (dashed orange line) at low temperatures may have been due to the thermally activated phase slips or finite-size effects [40]. Therefore, we determined that the difference in *T*_*c*_ values was due to the in-plane anisotropy rather than the films’ inhomogeneity. Remarkably, samples N3 and N4 showed the opposite behavior when the bridges with the higher critical current densities possessed a lower critical temperature, as shown in Figure 6b. 

Since the anisotropy was observed in the region of the phase fluctuations caused by thermally generated vortices, we considered two possible explanations of the superconducting transition anisotropy. The first is the anisotropy of a vortex motion and the second is an anisotropic Berezinskii–Kosterlitz–Thouless transition.

The description of the resistive transition in high-*T_c_* superconductors is quite complicated [41]. One of the simple explanations of the resistive transition in high-*T_c_* superconductors is a thermally activated vortex motion. Within the framework of Tinkham’s model of the resistive transition, a flux-flow resistance can be found as

*R_ff_ = R_n_ [I_0_ (U_0_/*2*k_B_T)]^−^*^2^*,*

where *R*_*n*_ is the normal-state resistance, *U*_0_ is the activation energy, *k*_*B*_ is the Boltzmann constant, and *I*_0_ is the modified Bessel function [26]. We found that our experimental *R*(*T*) curves are in good agreement with the predictions of Tinkham’s model, as shown in the Supplementary Materials. The anisotropy of the activation energy should result in the anisotropy of the flux-flow resistance. A higher critical temperature is expected for the bridges with the higher critical current density because of the activation energy *U*_0_~*J*_*c*_ [26]. The resistive transition anisotropy of the thinnest samples N1 and N2 and the thickest samples N5 and N6 were in good agreement with Tinkham’s model. In addition, the in-plane anisotropy of the vortex flow may have also originated from the anisotropic distribution of lattice defects such as grain boundaries.

However, samples N3 and N4 demonstrated a reversed sign of the resistive transition anisotropy when the bridges with higher critical current density had a lower critical temperature. This observation obviously cannot be explained by the in-plane anisotropy of the activation energy or the grain-boundary orientation because it does not depend on the film thickness. The reversed anisotropy of the resistive transition of the long anisotropic bridges may have been the result of the BKT transition.

The BKT transition is a transition between a low-temperature phase with logarithmically interacting vortices, which form bound states at low temperatures, and a high-temperature phase, where thermal fluctuations disrupt the bound states at temperatures above the BKT transition. In the BKT model, all thermally generated vortices are paired below the BKT transition temperature *T_BKT_*, resulting in zero resistance. The observation of the BKT transition is expected within a 2D superconductor with a size *l* greater than the effective magnetic penetration depth Λ of a thin film:

Λ = 2*λ*(*T*)^2^/*d*,

where *λ* is the magnetic penetration depth [42]. If the sample size is significantly smaller than Λ, vortex–antivortex pairs do not form [43]. For intermediate-sized samples, vortex–antivortex pairs and free vortices can coexist below *T*_*BKT*_ [44], and we assumed that the concentration of unpaired vortices and, thus, the resistance increase at *T* < *T*_*BKT*_ with a decrease in the *l*/Λ ratio.

In the case of long bridges, the BKT transition precondition is fulfilled in the longitudinal direction *L* > 2*λ*(*T*)^2^/*d* but may be broken in the transverse direction depending on the bridge width. Then, the resistive transition of long bridges with intermediate width is controlled by the *W*/Λ*_t_* ratio, where Λ*_t_* is the effective transverse magnetic penetration depth. Bridges of the same width and thickness but with different transverse magnetic penetration depths have different resistances at the same temperature within the range of the resistive transition.

Our anisotropic YTBCO bridges oriented in the a- and b-axis directions had critical current densities *J*_*ca*_ < *J*_*cb*_ and resistivities *ρ*_*ca*_ > *ρ*_*cb*_. Based on this, we may expect magnetic penetration depths *λ*_*a*_ > *λ*_*b*_ and, hence, *W*/Λ_*t*_ ratios in the transverse direction *W*/Λ_*b*_ > *W*/Λ_*a*_, respectively, for the same bridge width W. In the case of the BKT transition, the YTBCO bridges with lower critical current density must have a higher critical temperature compared to those with higher critical current density because they possess the greater *W*/Λ_*t*_ ratio in the transverse direction, resulting in the reversed resistive transition anisotropy. Therefore, the observation of the reversed in-plane anisotropy of the resistive transition in the anisotropic YTBCO films may be a new signature of the BKT transition in cuprate superconductors.

The observation of the BKT transition in cuprate superconductors is very challenging. While the broadening of the superconducting transition and the decrease in the zero-resistance temperature with a decrease in the film thickness, as well as the kinetic inductance jump predicted by the BKT model, have been consistently observed [44,45,46,47,48], evidence of the jump in the exponent *α* is inconsistent [49,50,51,52,53]. The inconsistency of the experimental data led to discussion on the existence of the BKT transition in the superconducting films. It was assumed that vortex pinning [54] or finite-size effects can mimic BKT behavior [55,56] or destroy it [43]. It was argued that attempts to observe the BKT transition could be unsuccessful due to inhomogeneity, improper sample size, or current noise [57]. Our YTBCO films have a smaller difference between a- and b-axis parameters compared to that of the pure YBCO films, which should lead to a smaller number of defects induced by the tetragonal–orthorhombic phase transition. Therefore, the reversed resistive transition anisotropy may indeed be due to the BKT transition.

**Figure 7:**
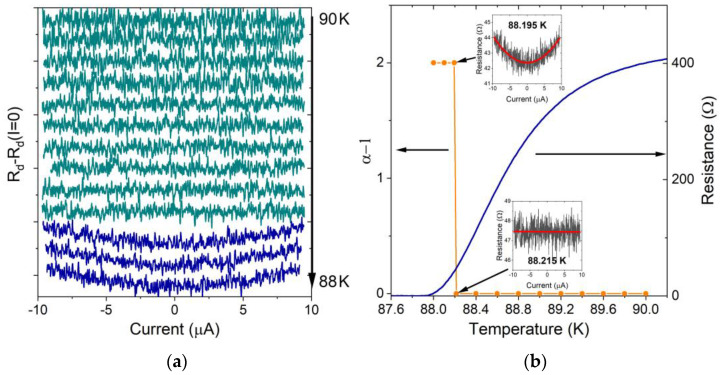
(**a**) The *R_d_*(*I*)-*R_d_* (*I* = 0) dependences of 19 μm wide and 30 nm thick YTBCO bridge in the 88–90 K temperature range. Curves are shifted along the ordinate axis for the convenience of their representation. Curves above and below the BKT transition are shown in dark cyan and dark blue, respectively. (**b**) The temperature dependences of the 19 µm wide and 30 nm thick YTBCO bridge resistance (dark blue line) and the *α*-1 exponent value (orange line). Inserts show *R_d_*(*I*) dependences at *T* = 88.195 and 88.215 K.

To confirm the existence of the BKT transition in the studied YTBCO bridges, we examined a hallmark of the BKT transition, namely the change in the exponent *α* in the current–voltage characteristic *V*~*I*^α^ from one to three at temperatures above and below the BKT transition [27]. The nonlinear behavior of the superconducting film at *T* < *T*_*BKT*_ is due to the current-induced unbinding of vortex–antivortex pairs. We measured the differential resistance *R*_*d*_ = *dV*/*dI* of the bridges with intermediate film thickness, where the occurrence of the BKT transition is suspected. Figure 7a shows a representative series of *R*_*d*_(*I*) − *R*_*d*_(0) dependences of the 19 μm wide and 30 nm thick bridge in the 88–90 K temperature range. The *R*_*d*_(*I*) dependences were measured within a ±9 μA bias current range to avoid the influence of the bias current on the resistive transition. We did not observe the signature of the Joule heating effect, which is characterized by an increase in the differential resistance of the bridge with increasing bias current at temperatures slightly below the onset of the superconducting transition, where the bridge resistance is high and the slope of the *R*_*n*_(*T*) curve is steep. Therefore, we ruled out the Joule heating effect at lower temperatures where the bridge resistance and corresponding Joule heating are smaller. The differential resistance demonstrated current-independent behavior at temperatures above 88.215 K and parabolic behavior at temperatures below 88.195 K which is a clear sign of the current-induced unbinding of vortex–antivortex pairs below the BKT transition temperature. The zoomed *R*_*d*_(*I*) curves at temperatures above and below the BKT temperature and the results of fitting the experimental curves by the *R*_*d*_ = const and parabolic dependencies are shown in the inserts in Figure 7b by black and red lines, respectively. The transition between the current-independent and parabolic *R*_*d*_(*I*) dependences was very sharp with a width below 20 mK. The temperature dependence of the *α* − 1 value and the resistive transition are shown in Figure 7b. Orange points indicate the temperatures where the *R*_*d*_(*I*) curves were measured. The vortex–antivortex pairing occurred at temperatures above the zero-resistance temperature, ruling out non-linearity caused by the vortex dynamics at currents above the critical current value. The BKT transition in the studied YTBCO bridges was not complete. Free vortices and vortex–antivortex pairs coexisted below the BKT temperature which might have been due to finite-size effects. In the case of the coexistence of vortex–antivortex pairs and free vortices, only the *α*−1 exponent in the *dV*/*dI*~*I*^*α*−1^ dependence shows the jump from 0 to 2 below the BKT temperature, as observed for our YTBCO bridges, while the change in the *α* exponent in the *V*~*I*^*α*^ dependence is continuous.

## 4. Conclusions

In conclusion, we fabricated high-quality YTBCO films with a very low surface roughness that exhibited in-plane anisotropy in both superconducting and normal states. We found that the increased resistivity and reduced critical current density were due to the reduced charge carrier density in YTBCO films compared to that in YBCO films, which is beneficial for high-kinetic-inductance devices. We observed a sharp switching of the *R_d_(I)* dependence of the YTBCO bridges in the resistive transition region from current-independent to parabolic behavior, which we consider to be a clear sign of the BKT transition. The reversed in-plane anisotropy of the resistive transition observed in the long YTBCO bridges could be explained within the framework of the BKT transition. We consider the YTBCO films to be promising for the fabrication of devices with high kinetic inductance and the investigation of the BKT transition in cuprate superconductors.

## Figures and Tables

**Table 1:** Parameters of the YTBCO samples including the film thickness *d*, the midpoint critical temperature *T_c,mid_*, the superconducting transition width Δ*T_c_*, the critical current densities *J_ca_* and *J_cb_*, and the normal-state resistivities *ρ_na_* and *ρ_nb_* in *a*- and *b*-axis directions, respectively.

N	*d*	*T_c,mid_*	Δ*T_c_*	*J_ca_* (77.4 K) *J_cb_* (77.4 K)	*J*_*cb*_/*J*_*ca*_	*ρ_na_* (100 K) *ρ_nb_* (100 K)	*ρ*_*na*_/*ρ*_*nb*_	*ρ*_*n*_ (300 K)/*ρ*_*n*_ (100 K)
	[nm]	[K]	[K]	[MA/cm^2^]		[µΩ·cm]		
1	11.8	87.7	1.9	1.25 ± 0.04 2.15 ± 0.13	1.72	111.3 ± 0.3 103 ± 1.06	1.08	2.4 ± 0.1
2	17	88.7	1.7	1.25 ± 0.05 2.48 ± 0.09	1.98	177 ± 5 122 ± 7	1.45	2.70 ± 0.03
3	21	90.1	2	5.09 ± 0.08 5.97 ± 0.09	1.17	91.5 ± 0.5 81 ± 2.4	1.13	2.92 ± 0.09
4	28	88.6	1.3	5.09 ± 0.15 6.07 ± 0.18	1.19	91.5 ± 0.4 85 ± 2.4	1.08	3.02 ± 0.03
5	35	89.7	0.7	5.41 ± 0.10 5.79 ± 0.27	1.07	89.3 ± 0.1 85.3 ± 0.1	1.05	3.03 ± 0.02
6	123	90.7	0.4	4.32 ± 0.03 5.32 ± 0.09	1.23	109 ± 3.5 99 ± 0.4	1.10	2.99 ± 0.04

## Data Availability

The data that support the findings of this study are available from the corresponding author upon reasonable request.

## Supplementary Materials

The following supporting information can be downloaded at: https://www.mdpi.com/article/10.3390/ma17030558/s1, Figure S1: Resistance temperature dependence of the 126 nm thick and 11.8 nm thick YTBCO bridges. Figure S2: Fitting of the resistance temperature dependences of the 19 μm wide YTBCO bridges with thicknesses of 123 nm and 11.8 nm by the Tinkham’s model.
