# Supplementary material for: In-Plane Anisotropy of Electrical Transport in Y0.85Tb0.15Ba2Cu3O7−x Films
Source: Materials (Basel). 2024 Jan 24;17(3):558. doi: 10.3390/ma17030558 (PMC10856356; doi:10.3390/ma17030558)
Supplement: Supplementary file 1 [file materials-17-00558-s001.zip › materials-2750599-supplementary.pdf]

SUPPLEMENTARY MATERIALS for

In-Plane Anisotropy of Electrical Transport in $Y_{0.85}Tb_{0.15}Ba_2Cu_3O_{7-x}$ Films

Matvey Lyatti[1,2,*], Ines Kraiem[1,2], Torsten Röper[1,2], Irina Gundareva[1,2], Gregor Mussler[1,2], Abdur Rehman Jalil[3], Detlev Grützmacher[1,2] and Thomas Schäpers[1,2]

[1] Peter Grünberg Institut (PGI-9), Forschungszentrum Jülich, 52425 Jülich, Germany

[2] JARA-Fundamentals of Future Information Technology, Jülich-Aachen Research Alliance, Forschungszentrum Juelich and RWTH Aachen University, 52425 Jülich, Germany

[3] Peter Grünberg Institut (PGI-10), Forschungszentrum Jülich, 52425 Jülich, Germany

* Correspondence: m.lyatti@fz-juelich.de

Temperature dependence of the YTBCO film resistance

The resistance *(R)* of the thick $Y_{0.85}Tb_{0.15}Ba_2Cu_3O_{7-x}$ (YTBCO) films followed a linear temperature (T) dependence (Figure S1a), while the resistance of the thinnest YTBCO films had a significant deviation from the linear dependence, as shown in Figure S1b. This deviation from the linear dependence became more pronounced when the YTBCO film thickness was reduced to a few unit cells. We attributed the deviation of the $R(T)$ dependence from the linear law observed for the thinnest films to the contribution of the few unit cell thick non-superconducting layer at the film-substrate interface, which has a semiconducting type of conductivity. The semiconducting layer contribution distorts the ratio between the resistance values at 100 and 300 K (R(300K)/R(100K)) which is frequently used to characterize the quality and the oxygen-doping level of the superconducting cuprate films. To obtain the contribution of the superconducting layer with the metallic type of the conductivity, we modelled the resistance of the film at the temperature above the critical temperature by a parallel connection of the resistors with the metallic ($A + BT$) and the semiconducting $C\cdot\exp(E_a/2k_BT)$ types of conductivity, where A, B, C, and E_a are constants, T is the temperature, and k_B is the Boltzmann constant. This simple model fitted the experimental curves well in the 120-300 K temperature range. At temperatures below 120 K, the experimental curve lay below the fitting curve because of the Aslamazov-Larkin fluctuation conductivity, which was not included in the model. The $R(T)$ dependences with and without the semiconducting layer contribution and the semiconducting layer contribution are shown in Figure S1b by black, red, and blue lines, respectively. To calculate the ratio R(300K)/R(100K), we used the $R(T)$ dependences with the contribution of the semiconducting layer subtracted. For the same reasons, we determined the in-plane anisotropy of the film resistivity at T = 100 K, where the contribution of the semiconducting layer is negligible.

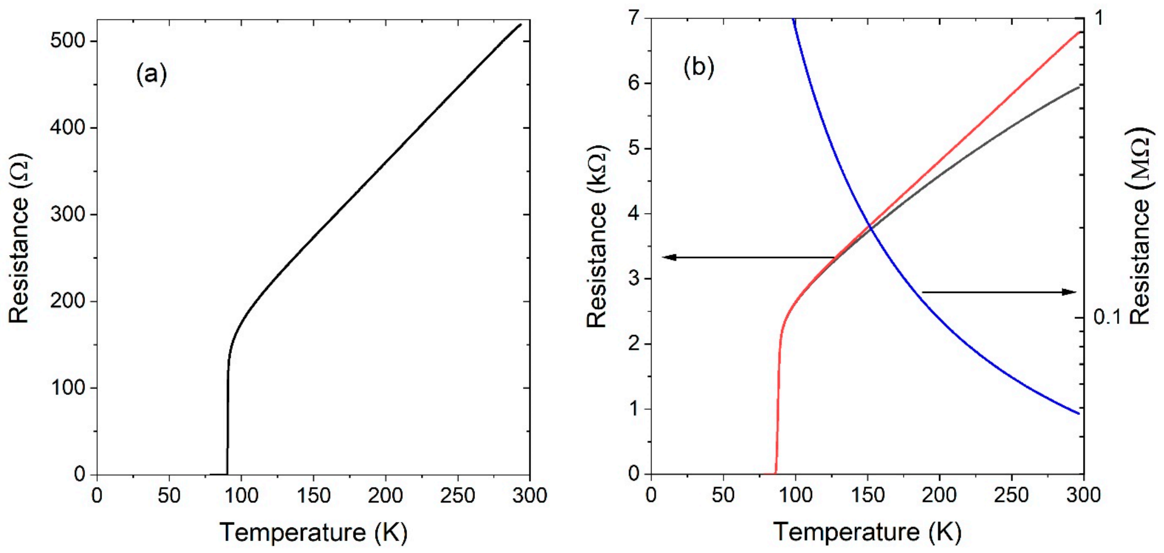

(a) (b)

Figure S1. Resistance temperature dependences of the 126-nm-thick (a) and 11.8-nm-thick (b) YTBCO bridges. The measured $R(T)$ dependence, the $R(T)$ dependence with the subtracted contribution of the semiconducting layer, and the semiconducting layer contribution are shown by black, red, and blue lines, respectively.

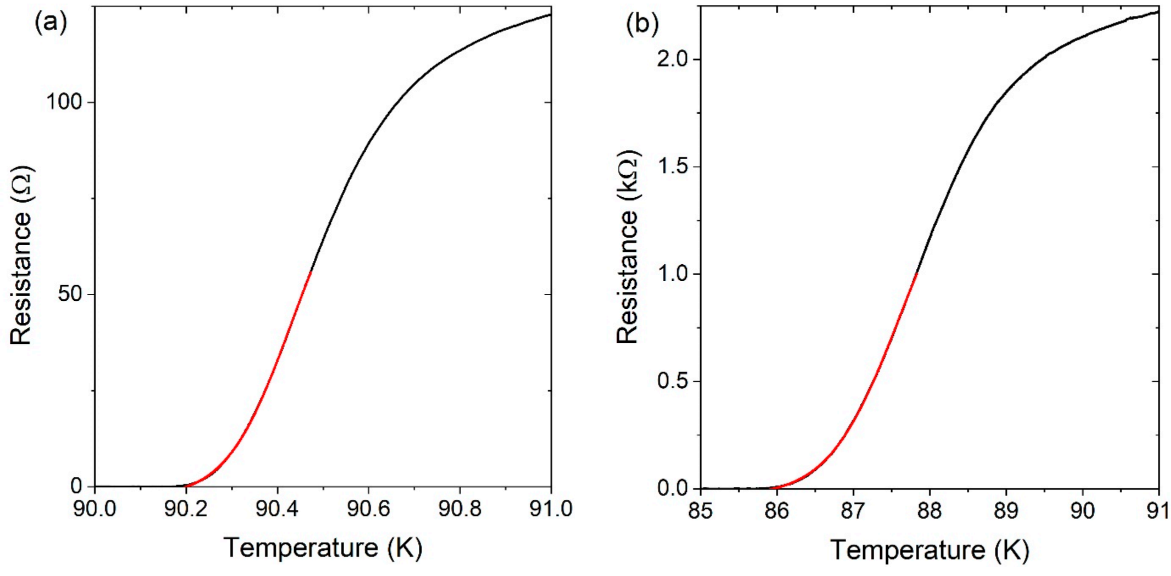

(a) (b)

Figure S2. Fitting of the resistance temperature dependences of the 19-μm-wide YTBCO bridges with the thickness of (a) 123 nm and (b) 11.8 nm (black lines) by the Tinkham's model (red lines).
